# PANDA e-health system as a tool to increase antenatal contacts and improve perinatal outcomes in Tanzania: adaptation and feasibility study

**DOI:** 10.1186/s12884-025-08263-y

**Published:** 2025-11-29

**Authors:** Valentina Actis Danna, Paschal Mdoe, Salum Ally, Sifael Katengu, Giovanna Stancanelli, Shangwe Kibona, Rose Laisser, Tracey Mills, Happiness Shayo, Andrew Weeks, Tina Lavender

**Affiliations:** 1 Centre for Childbirth, Women and Newborn Health, Department of International Public Health, Liverpool School of Tropical Medicine, Liverpool, UK; 2 Haydom Global Heath Research Centre, Haydom Lutheran Hospital, Manyara, Tanzania; 3 Terre Innovative Srl, Catania, Italy; 4 Catholic University of Health and Allied Sciences, Mwanza, Tanzania; 5 The Sanyu Research Unit, Institute of Life Course & Medical Sciences, Faculty of Health and Life Sciences, The University of Liverpool, Liverpool, UK

**Keywords:** MHealth, Antenatal, Tanzania, Feasibility, Quasi-experiment

## Abstract

**Background:**

Antenatal attendance is critical for pregnant women to receive the screening, health education and care plans that can optimise positive perinatal outcomes. In Tanzania, few women achieve the WHO-recommended 8 contacts with health workers, partly due to the limited screening and diagnosis services offered and health workers’ disrespectful attitudes. Mobile health solutions have potential to support the provision of comprehensive and respectful antenatal consultations, but these need to be rigorously assessed. The study aimed to adapt the Pregnancy and Newborn Diagnostic Assessment e-health system (PANDA), to incorporate respectful care prompts, and assess the feasibility of the new PANDA system to deliver prenatal care to women in rural Tanzania.

**Methods:**

A prospective, pre- and post-cohort mixed-methods study over 12 months was carried out in two primary facilities and one linked referral hospital. One hundred and sixty pregnant women (1st or 2nd trimester) were recruited; 80 received usual prenatal care and 80 received care through PANDA. Feasibility outcomes comprised recruitment and retention of women into the study, acceptability of the intervention and research processes, intervention fidelity, feasibility of data collection, confirmation of trial outcome measure and quality of implementation.

**Results:**

Incorporation of respectful care prompts was achieved and considered acceptable. The study recruited to target (> 90%) in both groups and 100% of women were retained in the study. Fidelity of the PANDA package, including training, was confirmed through observations and clinical data. Two interlinked themes summarised the qualitative narratives: *PANDA supports relational care*, and *PANDA enables systematic and personalised ANC provision.* PANDA was considered a positive intervention, although its use prolonged the first consultation duration.

**Conclusion:**

Acceptability of PANDA use and feasibility of study processes were demonstrated. Participants’ and stakeholders’ feedback have informed refinement of the PANDA package and its implementation strategy ahead of commencing a cluster trial to assess effectiveness.

**Trial registration:**

The study was prospectively registered (ISRCTN34645009) on 21/10/2022.

## Background

Accessing antenatal care (ANC), throughout pregnancy, is associated with improved pregnancy outcomes [1, 2]. Several studies have demonstrated an increased risk of perinatal death among women who did not attend ANC [3, 4], had less than four ANC visits or a later initiation [1, 5–7]. However, women’s uptake is often less than recommended because of deterrents, including negative past experience, disrespectful care, difficulties accessing transport, long waiting times, lack of privacy during visits and poor environment [8]. Additionally, ANC visits are often task-orientated and fail to accommodate women’s childbirth choices [9]. Women value ANC services that they perceive to be beneficial to them and their baby and that are provided by friendly and respectful health workers [10]. Mobile health, or mHealth, solutions have been offered as a means to deliver effective, supportive and accessible service.

MHealth, a component of electronic health (eHealth), refers to the use of an electronic device, including smartphones, to support and deliver healthcare contents and services [11, 12]; the use of smartphones use has rapidly increased in recent years in Low and Middle-Income countries (LMICs). In 2023, around 4.3 billion people, more than three quarters of the global population, were phone owners [13] making this technology integral to people’s lives.

Within maternal and newborn health (MNH) service delivery, mHealth has shown several benefits. Two recent studies [14, 15] highlighted how mHealth has enabled healthcare workers to collect real-time data on maternal and perinatal health and use the data to make informed decisions about potential complications and identified risks. This data can also be used at district and regional levels for monitoring and evaluation purposes, improving the overall effectiveness of healthcare system delivery [15]. MHealth solutions often include educational and behaviour change elements to support better maternal and perinatal outcomes, for example, prompts to attend checkups, advice regarding birthing in facilities, and complication-readiness training [15]. MHealth has also targeted health workers to minimise errors in patient records and facilitate communication with clients [16].

The Pregnancy and Newborn Diagnostic assessment e-health system (PANDA) [17] is an mHealth solution with potential to support ANC in LMICs. PANDA draws on WHO’s ANC recommendations and contents [18], with the aim of facilitating high-quality, comprehensive and standardised ANC visits. Since release in 2014, PANDA has been used in Italy [19], Madagascar [20], Tanzania [21] and Burkina Faso [22], with varying levels of evaluation. The application was piloted with 150 pregnant asylum seekers, in Italy, and demonstrated acceptability of antenatal visit duration and health education content [19]. Women’s satisfaction index was high; however, caregivers’ experience was not evaluated. In Madagascar [20], PANDA enabled the creation of individual electronic medical records for 100 pregnant women. The in-built system of flagging abnormal results facilitated referral and follow-up in subsequent visits. Women and healthcare workers were satisfied with PANDA, although satisfaction measures were not clearly explained. In southern Tanzania, a nested study [21] within a non-randomised intervention study surveyed acceptability of PANDA among 98 women (52 in the intervention group; 46 in the control group). The majority of participants (96.2%) who received care through PANDA found the visit length adequate, would return for subsequent visits and would recommend ANC to a relative or a friend. Focus group discussions (FGDs) confirmed women’s satisfaction, and highlighted appreciation of birth preparedness, and pictorial communications. In the same study, health workers’ self-reported questionnaires confirmed acceptability of PANDA use, although the sample size was small (*n* = 5). In Burkina Faso [22], 591 women (319 in intervention, 272 in control groups) were enrolled in a cluster randomised controlled trial evaluating the effectiveness of PANDA in improving the quality of ANC, using an author-developed score derived from 6 components: welcoming the woman, history taking and dietary habits, physical examination, obstetric examination, preventive care, and counselling and advice [22]. Although PANDA improved the ANC quality, when compared to the control group, the quality rating remained low overall (15.67% vs. 6.25%). Results were attributed to the composite nature of the ANC quality score and the overall poor quality of ANC in some LMICs [12]. None of the studies to date have investigated the effectiveness of PANDA use in improving clinical outcomes. Furthermore, the impact of PANDA use on respectful care remains unclear. Respectful care, is a priority consideration for women when choosing to engage with ANC services [13].

Thus, we conducted a study to adapt PANDA to incorporate respectful care elements and assess the feasibility of conducting a large effectiveness trial to increase antenatal contacts and improve perinatal outcomes in Manyara region, Tanzania.

## Methods

### Design and study setting

The study was informed by the MRC/NIHR framework for development and evaluation of complex interventions [23]. This framework separates complex intervention research into four phases: intervention development, feasibility, evaluation, and implementation [23]. Core to these elements is consideration of contextual factors, engagement with local communities and other key stakeholders, and refinement of interventions prior to evaluation and economic considerations. We report on the development and feasibility phases.

The developmental phase comprised an observational qualitative study to determine the intervention components. The feasibility phase comprised a prospective mixed-methods cohort study using a pre- and post-observational design. The study was part of the NIHR Unit on Prevention and Management of Stillbirth and Neonatal Death in Sub-Saharan Africa and South Asia (NIHR 132027). The COREQ [24] and CONSORT checklist extension [25] were used to report the qualitative and feasibility results, respectively.

The study was conducted in Tanzania, which bears a high-burden of perinatal deaths, with an estimated 40,480 stillbirth and 42,814 neonatal deaths annually, corresponding to a stillbirth rate of 18.8 per 1000 birth and a neonatal mortality rate of 20.1 per 1000 live births, respectively [26]. About 77% of stillbirths and almost 60% of newborn deaths occur to women living in rural areas with a considerable proportion (20%) belonging to the lowest level wealth quintile [27]. Thus, a rural region in North-East Tanzania was chosen, and included two dispensaries and one linked referral hospital. Dispensaries were chosen for their volume of ANC bookings (at least 50 per month), availability and willingness of nurse-midwives to participate, and proximity to the referral hospital (within 20 km).

### Study intervention: PANDA mHealth system

**Fig. 1:**
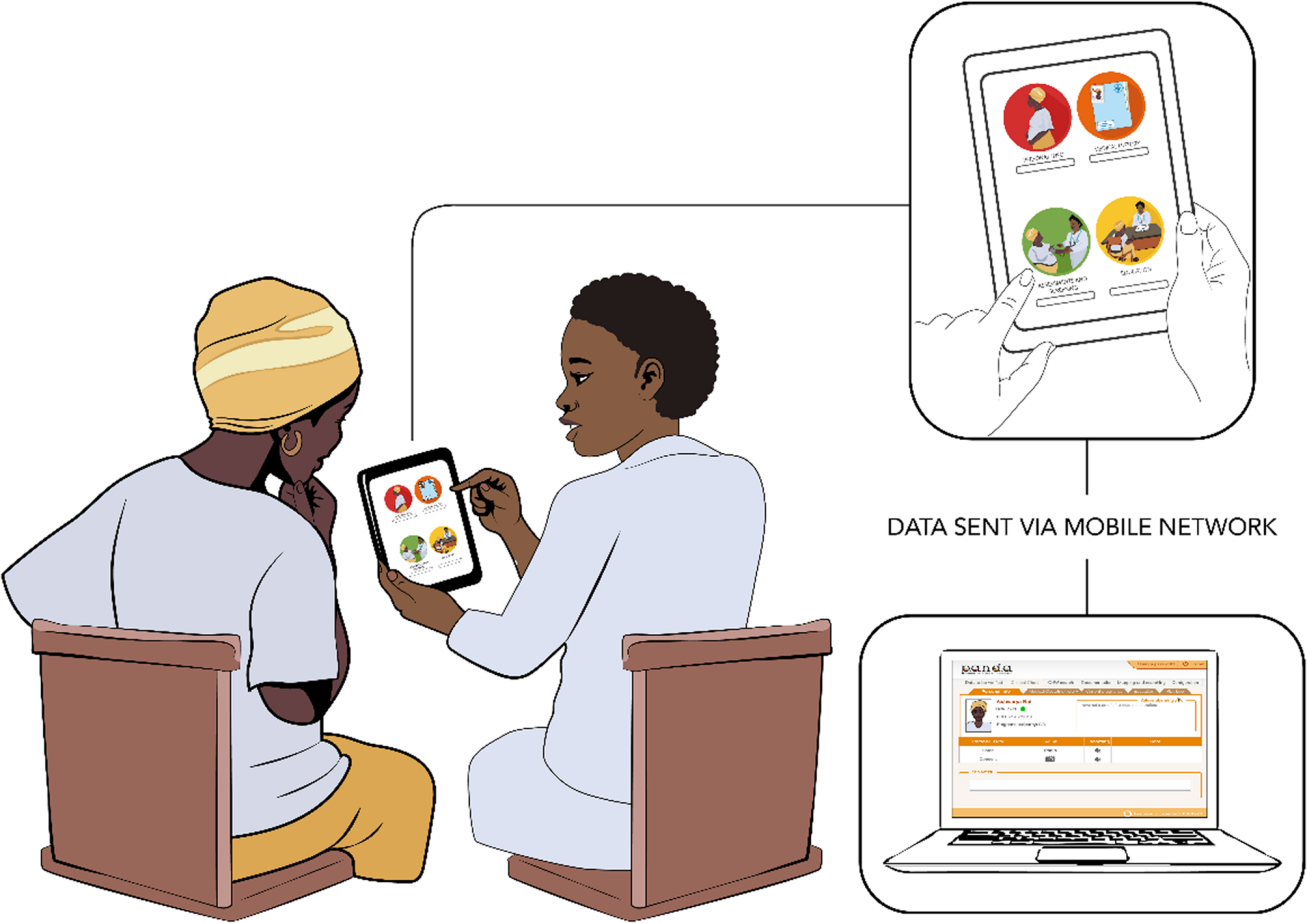
Panda e-Health System

PANDA is a telemedicine system used by healthcare workers to deliver ANC and includes three components: a smartphone application, point of care testing, and a Medical Unit database (Fig. 1). It is an android-based application usable on smartphones or tablets and structured in four modules: personal information, medical history, assessment and screening and health education (Fig. 1).

Through the application, the woman’s details and obstetric history are recorded by the healthcare worker, and her current pregnancy is screened and assessed to identify risks and possible complications. The health education module supports provision of information about well-being and hygiene during pregnancy, birth preparedness and complication readiness, and essential newborn care. PANDA aims to support the provision of comprehensive and respectful antenatal care, thus encouraging women to have the motivation to attend the clinic.

#### Process

At the first antenatal appointment the nurse-midwife creates a new clinical record and includes an ANC number and a barcode. A copy of the barcode is attached to the woman’s antenatal card to facilitate retrieval of information at subsequent appointments. The nurse-midwife is prompted to follow the modules in sequence, complete the consultation and “close the visit”. If internet connection is available, the application automatically sends the record to the Medical Unit database. When the woman attends subsequent appointments, the provider retrieves her record either through her ANC number or the barcode. Alert messages appear automatically to flag abnormal results and to prompt action. *Point of care testing* comprised a set of diagnostic tools and tests. Resources were provided to the dispensaries at the start of the intervention phase and included electronic blood pressure machines, weighing scales, height measurement devices, electronic thermometers, urinalysis sticks, rapid tests for HIV, malaria and syphilis and cuvettes to measure haemoglobin level.

*The medical unit component* consists of a web-based, password protected database of antenatal records of the women attended using PANDA. Records were received from the PANDA application via the internet and could be downloaded and reviewed offline. The system automatically classified women based on risks identified during the consultation. Daily validation of the visits, by an obstetrician, included confirming the risk identified in the system and adding any new ones. Notes on actions for subsequent visits were also added.

### Developmental phase (Adaptation in context)

Adaptation of PANDA involved stakeholder engagement, non-participant observations, and focus group discussions (FGDs), over a 6-month period.

A working group (*n* = 11), comprising senior clinician researchers (*n* = 5), research assistants/associates (*n* = 5) and a technical expert (*n* = 1) adapted PANDA for the Tanzania context by reviewing app contents against Tanzanian ANC Providers’ Guidelines [28, 29] and WHO recommendations [18]. Once a prototype was agreed, feedback on content and implementation was sought from a stakeholder group (*n* = 14) comprising Ministry of Health representatives, Regional and District Medical and Nursing officers, District coordinators for Reproductive and Child Health services, IT experts from the Regional and District councils, facility representatives and a Community Engagement and Involvement group (CEI) comprising women who had accessed maternity services in the region. Stakeholders’ suggestions resulted in translation of the application into Swahili, and inclusion of three question related to mental health (past history, current illness and family history), with an alert message for women at high-risk. They also informed the choice of rural, remote sites to benefit the most vulnerable population. One expressed concern was connectivity in the region. This resulted in adoption of a hybrid approach whereby data was collected offline, and synchronisation done later where there was a stable connection. The CEI group recommended word changes for understandability, and ensured the pictorial design was intuitive and culturally appropriate (e.g. clothes worn by women).

Non-participant observations (*n* = 6, three per site) and focus group discussions (FGDs) (*n* = 6) with health workers (*n* = 24) and women (*n* = 25) informed PANDA adaptation. Data collectors were all midwives (male and female) and were employed and trained as research assistants.

**Fig. 2:**
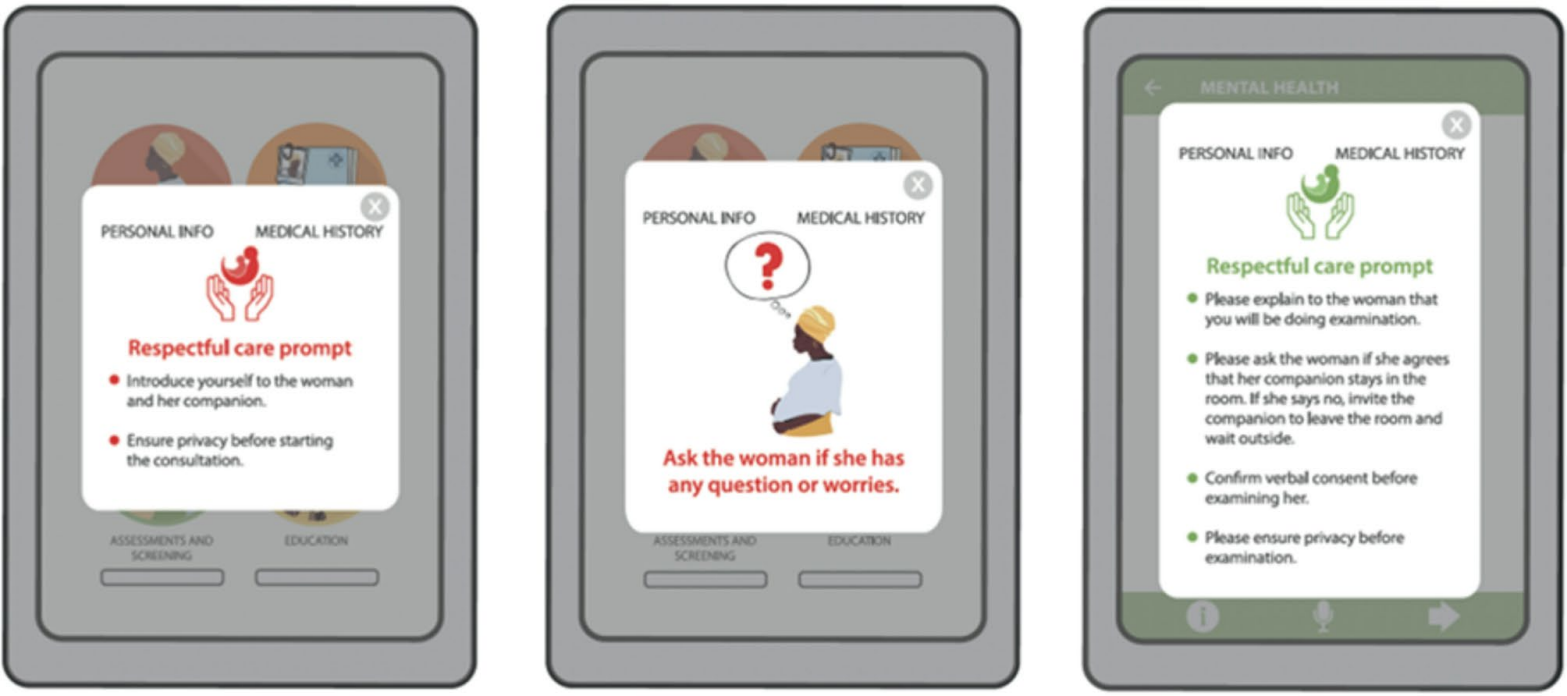
Respectful care prompts

Observations were used to explore context, processes, behaviours and communication between health workers and women during provision of usual ANC. Participants were purposively identified and approached by a research midwife, who provided informed consent for the consultation to be observed. No personal details were recorded. The observation followed a three stages approach [30], and was used to visualise nuances of people’s interaction and communication [31]. A template, adapted from previous studies [32], supported note-taking. To minimise the Hawthorne effect, observations were non-obtrusive and carried out throughout the day and on different days. Observations enabled images of the local settings to be relayed to the graphic designer to align PANDA images to the context. Observations also identified the lack of respectful care, which informed the inclusion of fifteen health worker respectful care prompts within the app (Fig. 2); features not previously included. These prompts acted as reminders to provide respectful care and included (1) explaining an examination or assessment to the woman (Blood pressure, screening tests, breast examinations) (2) asking for verbal consent before a procedure, (3) maintaining privacy, (4) seeking consent for a companion to be present, (5) explaining the results of tests and (6) offering the woman opportunities to ask questions or express concerns.

FGDs (*n* = 3 for each group) explored health workers’ and women’s acceptability of PANDA contents and layout, practicality of use and implementation. A maximum variation recruitment strategy was used to create a heterogenous group of participants who could share different perspectives and experiences. Health workers were purposefully chosen for their role and years of experience providing ANC. Pregnant women comprised a mixture of primigravida and multigravida, with or without living children and at different stages of pregnancy (12 to 36 weeks gestation). A topic guide, with open questions, explored views on the PANDA design, the structure and sequence of contents, implementation and useability. Before the discussion, participants were given time to review the PANDA tablet and PANDA manual (comprising step-by-step guide of PANDA fields). FGDs, conducted in Swahili, were dual-moderated and took place at a convenient and safe community venue. Interviews were audio-recorded, transcribed verbatim, translated and back-translated, prior to being subjected to framework analysis [33]. Focus group discussions were conducted with 49 participants, three with healthcare workers and three with women (Table 1). No participants had engaged with mHealth previously. Focus groups included eight or nine participants each and lasted between 39 and 92 min.

**Table 1:** Demographics of participant involved in the FGD

Health workers (*n* = 24)		
Age, median [IQR]		36 [28–52]
Sex	Male	6
	Female	18
Education	Primary/Secondary	3
	Certificate/Diploma	12
	Degree	9
Job position	Nurse-midwife	9
	Doctor	8
	Medical assistant	6
	Other	1
Years of work experience, Median [IQR]		13.5 [3–25.5.5]
Years of experience in maternal care including ANC, Median [IQR]		10 [2–17]
Women (*n* = 25)		
Age (years)	Median [IQR]	26 [21–31]
Area of residence	Rural	23
	Urban	2
Civil status	Married/Living with partners	22
	Single/Separated-divorced	3
Education	Never been to school	3
	Primary	17
	Secondary or higher	5
Occupation	Homemaker	16
	Self-employed	7
	Formally employed	2
Religion	Christian	24
	Muslim	1
Tribe	Iraq	21
	Other	4
Primipara	(N, %)	7
N living children, Median [IQR]		3.50 [2–5]
ANC visits in last pregnancy, Median [IQR]		4.50 [3–5.75.75]
Gestational age at booking, Median [IQR]		20 [12–24]
Gestational age at time of FGD, Median [IQR]		30 [24–35]

Health workers and women welcomed the PANDA application and appreciated its contents and design, and provided feedback related to five areas: language, design, ANC visit length, loss of data and prompting respectful care. Supporting quotes are provided as examples of participants’ responses and the recommendations for the feasibility phase highlighted (Table 2).

**Table 2:**
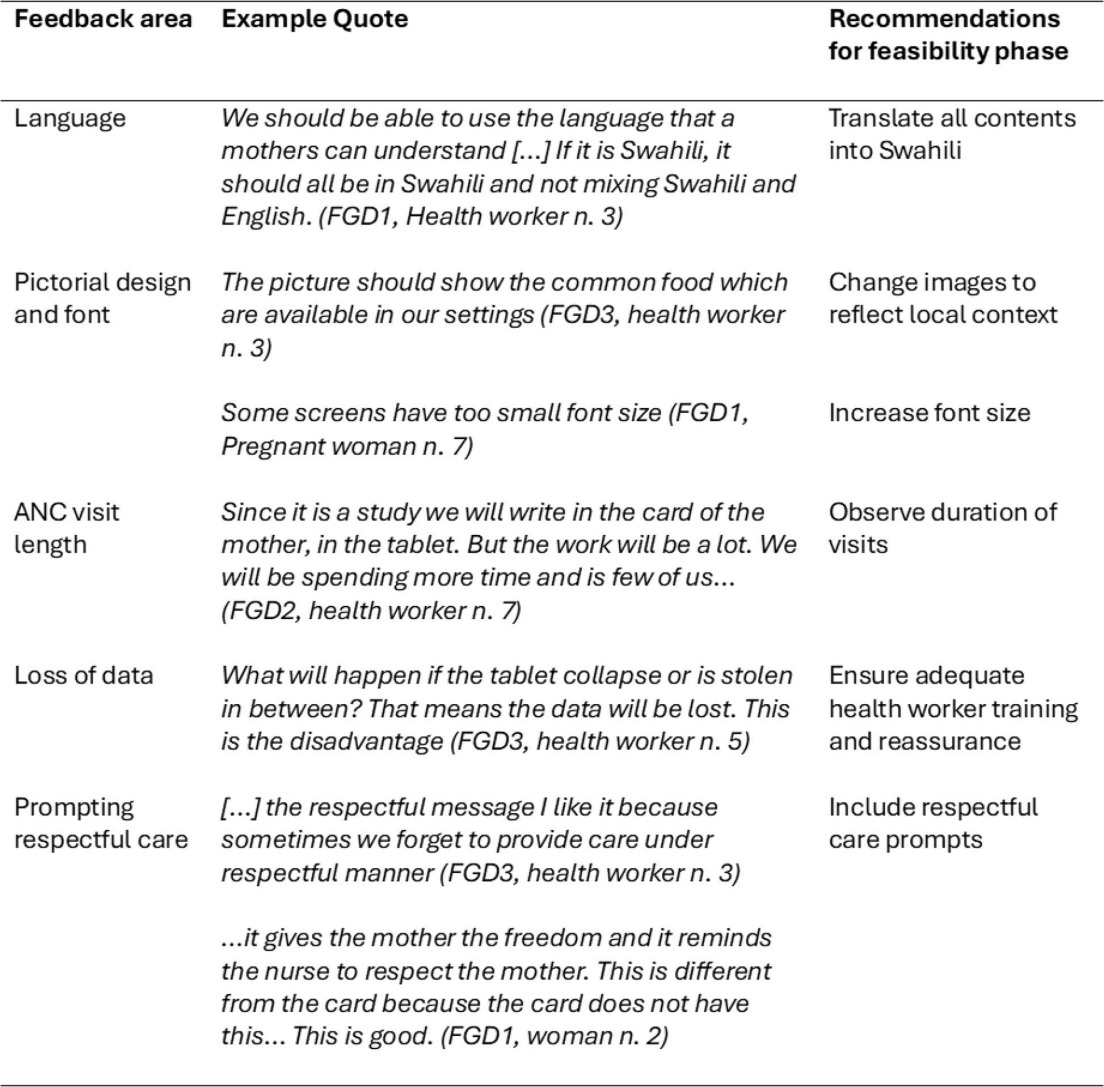
Focus group responses and feasibility recommendations

### Feasibility phase: methods

Following the developmental phase, in which PANDA was adapted, a prospective mixed-methods study was conducted over a 12-month period. A pre- and post-cohort design was used for implementation of the revised intervention and assessment of feasibility. Feasibility outcomes comprised study recruitment and retention rates, acceptability of the intervention and research processes, intervention fidelity, feasibility of data collection, confirmation of trial outcome measure and quality of implementation.

### Study sample and recruitment

One hundred and sixty women (40 per site, per phase) and six health workers were recruited from the two dispensaries. Recruitment did not take place in the referral unit; this site was included to house the medical unit (described later). The sample size was determined pragmatically in line with the accepted criteria for feasibility studies [34], which enabled implementation of the intervention in two sites and an estimate of recruitment and retention rates. Eligible women were ≥ 18 years, with a low-risk pregnancy, and were booked or planning to book ANC at the study facilities. A non-probability sample of all eligible women who attended study sites during a set period of time were included. In phase one (pre-intervention) 80 women were recruited and offered the usual ANC; in phase two (intervention) a further 80 women received care through PANDA. Women were recruited over a 4–6 week period. Workshops held at the dispensaries informed the staff about the study. Posters were placed in client-facing areas in the two facilities. While waiting for their antenatal consultation, eligible women were identified and approached by a health worker who provided a brief introduction to the research. Interested women were given additional information by the research team and, if willing to participate, written consent obtained at this initial contact. This consenting strategy was used for two reasons: firstly, some women only attend one visit [35] therefore delaying consent may prevent their participation. Secondly, it would be unreasonable to request women to return to the facility for research purposes, due to impact on their time and resources. However, women were encouraged to discuss participation with their families and consent was reaffirmed at subsequent appointments, to enable participants to withdraw if they wished to.

### Outcome measures

The primary outcomes for feasibility were recruitment and retention of women in the study. Recruitment targets had to be met and ≥ 70% women retained into the study for the trial to be considered feasible. Secondary outcomes included acceptability of ANC with the aid of PANDA and experiences of study processes and data collection tools.

### Data collection

The research team kept screening and recruitment logs for all women invited to participate in the study, including details about reasons for non-recruitment, withdrawal and those lost to follow-up. Pre- intervention group data were extracted from the ANC registers and put into an electronic case report form (CRF) built in Research electronic data capture (REDCap) [36]. CRFs included demographic information, previous obstetric history, medical conditions, vaccination, screening for Syphilis, HIV and Malaria, records of blood pressure, haemoglobin level, fundal height, albumin in urine and physical examination details. Intervention group data, including demographics, obstetric details and results from assessments and screening, were automatically collected through PANDA which created an electronic medical record for each woman. After the birth the research team contacted and consented the woman to complete three questionnaires: Quality of Prenatal Care Questionnaire [37] a 46-item validated tool, which was formally translated into Swahili; Person Centred Prenatal Care Scale [38] a 34-item validated tool for assessment of respectful care components, and an investigator-designed exit questionnaire exploring acceptability of the research tools and PANDA, and experience of taking part in the study. Completion of CRFs occurred at the facility or in the woman’s preferred venue, or via telephone and was either self-directed or researcher-administered depending on the woman’s literacy level. A sample of women (*N* = 12 control group and *n* = 11 intervention group) and health workers (*n* = 5) were also invited for a semi-structured one-to-one interview during the same follow up visit (women) or at the end of the study (health workers). Interviews, carried out by research assistants who were also registered midwives, were conducted in Swahili, face-to-face, audio-recorded, translated and transcribed verbatim.

### Data analysis

Quantitative data, collected in REDCap database and PANDA database were imported and analysed in SPSS v. 26. Demographics, clinical and outcome data for control and intervention groups were compared descriptively, using frequencies and percentages for categorical variables and descriptive statistics, including means, standard deviations, median and ranges for numerical variables. Questionnaire scores (QPCQ and PCPS) were calculated and rates of missing data compared.

Qualitative data gathered from observations, FGDs and interviews were analysed using the Framework approach (33), enabling a deductive and inductive approach. Five stages were followed including data familiarisation, coding, indexing, charting, mapping and interpretation. During familiarisation two researchers (VAD, SK) immersed themselves in the data and identified main ideas and concepts, which were then organised into a theoretical framework. With support from other researchers (HS, MS, SA) this framework was then reapplied to the raw data and refined as needed. Data were then summarised into thematic charts and reviewed by the wider research team to confirm the overall interpretation and authenticity of key messages.

## Results

### Recruitment and retention

A total of 160 participants took part in the feasibility study. In both pre intervention (usual care) and intervention phases, the recruitment target of 80 women (*n* = 40 per facility) was met (Table 3).

**Table 3:** Recruitment and retention

Recruitment		Control	Intervention	Total
Approached		86	84	170
Not eligible (% approached)		1 (1.2%)	2 (2.4%)	3 (1.8%)
Eligible		85	82	167
Decline (% eligible)		5 (5.9%)	2 (2.4%)	7 (4.2%)
Reasons for declining	No reason given	4	1	5
	No permission from husband	1	1	2
Recruited (% eligible)		**80 (94.1%)**	**80 (97.6%)**	**160 (95.8%)**
Retention				
Completed study (% recruited)		80 (100%)	80 (100%)	160 (100%)

*NB* Data are n (% of total per group) unless indicated

**Fig. 3:**
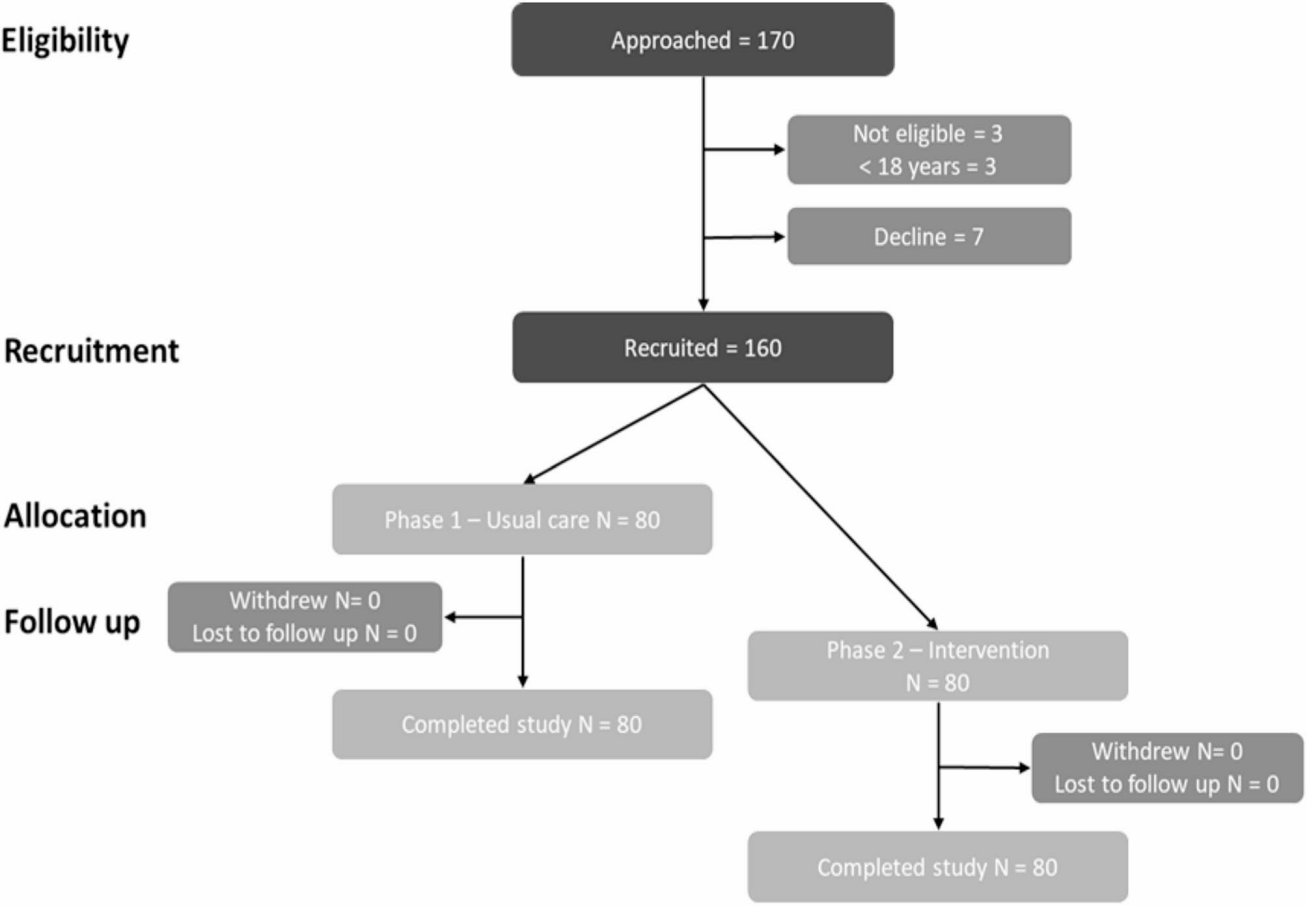
– Participants’ flow

Recruitment for the pre intervention group took five weeks; 86 women were approached; one was not eligible due to age < 18 years. Among the eligible sample (*n* = 85), five women declined participation, four did not provide a reason, and one was prevented from taking part by her husband. Recruitment of the intervention group took eight weeks; 84 women were approached and two were not eligible. Of the 82 eligible women, one did not supply a reason for declining, and the other was not given permission from her husband. All recruited women were followed up post-birth and completed the study, providing 100% retention rate. However, some women did not have phone numbers, resulting in researchers following women up via community health workers and village leaders. The participant flow is shown in Fig. 3. There were 25 adverse events; none were related to the intervention.

### Participants

Participants’ characteristics are presented in Table 4, alongside the target population. The mean age of women was 26 years (SD = 6.2) and was similar across phases. The majority of women were married or living with a partner (93.1%) and completed primary education (86.3%). In both phases, more than three quarters of women were farmers (87.5%). Almost a quarter of participants were experiencing their first pregnancy while those who had previous pregnancies had an average of two living children [SD = 2.14]. Study participants had similar characteristics as the target population.

**Table 4:** Participants’ demographics, pregnancy history, antenatal care attendance and birth outcomes

		**Target population**	**Control **	**Intervention**	**Total **
Participants (n)		366	80	80	160
Age (years; mean [SD])		26.3 (6.2)	25.6 (6.5)	26.2 (5.9)	25.8 (6.2)
**Civil status**					
-Single			5 (6.3%)	6 (7.5%)	11 (6.9%)
-Married / Living with partner			75 (93.8%)	74 (92.5%)	149 (93.1%)
**Education**					
-Primary			73 (91.3%)	65 (81.3%)	138 (86.3%)
-Secondary or higher			7 (8.8%)	15 (18.8%)	22 (13.8%)
**Occupation**					
-Homemaker			4 (5.0%)	6 (7.5%)	10 (6.3%)
-Farmer			74 (92.5%)	66 (82.5%)	140 (87.5%)
-Self-employed			2 (2.5%)	5 (6.3%)	7 (4.4%)
-Formally employed			-	1 (1.3%)	1 (0.6%)
-Student			-	2 (2.5%)	2 (1.3%)
**Pregnancy history**					
First pregnancy		72 (19.7%)	18 (22.5%)	20 (25%)	38 (23.8%)
N. of living children Mean (SD)		2.32 (2.11)	2.34 (2.27)	2.10 (2.00)	2.22 (2.14)
**Antenatal care**					
Gestational age at booking (weeks), median, [IQR])		20 [16-26]	20 [17-24]	24 [20-27]	22 [19-26]
N. of ANC visits attend in last pregnancy, median [IQR]			4.0 [3-7]	4.0 [3-7]	4.00 [3-7]
< 4 visits			23 (28.7%)	27 (33.8%)	50 (31.3%)
4 visits			24 (30.0%)	25 (31.3%)	49 (30.6%)
> 4 visits			14 (17.5%)	4 (5.0%)	18 (11.3%)
Not applicable			19 (23.8%)	20 (25.0%)	39 (24.4%)
N. of ANC visits in current pregnancy, median [IQR]		3.0 [2-4]	4.0 [3-5]	4.0 [3-5]	4.0 [3-5]
< 4 visits			27 (33.8%)	27 (33.8%)	54 (33.8%)
4 visits			22 (27.5%)	30 (37.5%)	52 (32.5%)
> 4 visits			31 (38.7%)	23 (28.7%)	54 (33.8%)
**PANDA (intervention group only)**					
N. ANC visits with PANDA, median [IQR]		N/A	N/A	2.0 [1-3]	N/A
ANC visit duration (min, mean [SD])		N/A	N/A	18:08 (10:31)	N/A
First visit (min, mean [SD])		N/A	N/A	25:07 (08:38)	N/A
**Complications during pregnancy**					
No		363 (99.2%)	67 (83.5%)	76 (95.0%)	143 (89.4%)
Yes		3 (0.8%)	13 (16.3%)	4 (5.0%)	17 (10.6%)
-Proteinuria		-	-	1 (25%)	1 (5.9%)
-PVB		-	1 (7.7%)	-	1 (5.9%)
-Fibroid in uterus		-	5 (38.5%)	-	5 (29.4%)
-Oedema		-	2 (15.4%)	2 (50%)	4 (23.5%)
-Vomiting, low BP		2 (66.6%)	2 (15.4%)	1 (25%)	3 (17.6%)
-Abdominal pain		1 (33.3%)	2 (15.4%)	-	2 (11.8%)
-No fetal movement		-	1 (7.7%)	-	1 (5.9%)
**Birth outcomes**					
-Livebirth			77 (96.3%)	78 (97.5%)	155 (96.9%)
-Stillbirth			3 (3.8%)	2 (2.5%)	5 (3.1%)
Mode of Birth					
-SVD			79 (98.8%)	71 (88.8%)	150 (93.8%)
-C-section			1 (0.3%)	9 (11.3%)	10 (6.3%)
**Place of birth**					
-Dispensary			20 (25.0%)	23 (28.7%)	43 (26.9%)
-Referral hospital			25 (31.3%)	25 (31.3%)	50 (31.3%)
-Home			33 (41.3%)	30 (37.5%)	63 (39.4%)
Born Before Arrival			2 (0.5%)	2 (2.5%)	4 (2.5%)

Participants booked their ANC in the second trimester. 66% of women attend four or more visits, the national guidelines’ recommended number, in both phases. However, for women who had a previous pregnancy, attendance of four or more visits during this research, was higher than in the previous pregnancy (66.3% vs. 42.9%). The intervention group had a median of two antenatal care visits delivered through PANDA with a mean duration of 18 min. In contrast, standard care visits lasted between eight to ten minutes as observed during the development phase. Birth outcomes were similar in phase one and two, more women birthed by caesarean section in phase 2 (11.3% vs. 0.3%). More than a third of women gave birth at home.

### Intervention implementation

PANDA e-Health system was introduced towards the end of the control phase, when almost all women had given birth, to limit contamination between study phases. Four nurse-midwives were recruited from the study sites following information seminars and agreement with the facility manager. A one-day interactive training workshop was held in Swahili at the referral facility by the Tanzania research team. Training included an introductory session to the PANDA system, hands-on PANDA practice and a Q&A session. Formal classroom-based sessions were followed by on-the-job training provided by the research team at the facility, during real-time use. Researchers ensured that health workers knew how to register a participant with the barcode, open and close a visit, conduct subsequent visits and check that data had synchronised with the medical unit. A specific training session, on how to review and validate visits in the medical unit, was also delivered to two senior obstetricians in the referral hospital. Following the training, the research team distributed point of care supplies to both dispensaries, and the staff started using PANDA with women for first and subsequent visits. Data were regularly synchronised to the medical unit and validated on a weekly basis.

### Outcomes and acceptability

During the feasibility study we compared the maternal screening schedule against the Tanzanian guidelines. Overall, women in the intervention group received more screening than in the control group (Table 5).

**Table 5:** Measurement and screening during antenatal consultations

	Antenatal care visit numbers					
Control	I	II	III	IV	V	VI
Sample attending	80	78	68	53	30	10
Blood pressure check	52 (65.0%)	47 (58.8%)	42 (52.5%)	26 (32.5%)	17 (21.3%)	6 (7.5%)
Haemoglobin check	3 (3.8%)	1 (1.3%)	3 (3.8%)	4 (5.0%)	2 (2.5%)	1 (1.3%)
HIV test	68 (85.0%)	7 (8.8%)	9 (11.3%)	3 (3.8%)	-	-
Malaria test	66 (82.5%)	5 (6.3%)	5 (6.3%)	3 (3.8%)	-	-
Syphilis test	21 (26.3%)	1 (1.3%)	-	1 (1.3%)	-	1 (1.3%)
TB screening	-	1 (1.3%)	-	-	-	-
Intervention	I	II	III	IV	V	VI
Sample attending	80	75	64	53	23	6
Blood pressure check	60 (75.0%)	63 (78.8%)	56 (70.0%)	46 (57.5%)	20 (25.0%)	6 (7.5%)
Haemoglobin check	36 (45.0%)	26 (32.5%)	34 (42.5%)	36 (45.0%)	16 (20.0%)	5 (6.3%)
HIV test	78 (97.5%)	1 (1.3%)	2 (2.5%)	4 (5.0%)	2 (2.5%)	1 (1.3%)
Malaria test	76 (95.0%)	2 (2.5%)	1 (1.3%)	2 (2.5%)	2 (2.5%)	-
Syphilis test	73 (91.3%)	2 (2.5%)	1 (1.3%)	5 (6.3%)	2 (2.5%)	-
TB screening	8 (10.0%)	-	1 (1.3%)	1 (1.3%)	-	-

*Numbers and percentages refer to women who had the screening test or check done at each visit

Over half of all women had their blood pressure (BP) assessed on at least three visits. In the intervention group over 50% of participants had their BP assessed during the fourth visits, however, fewer women in both groups had BP assessments at the fifth and sixth contacts. Haemoglobin (Hb) screening was conducted in less than 5% of women attending visits pre intervention, with higher rates observed in the intervention group (20–40%). HIV and Malaria testing were similar in both groups. Syphilis testing was completed for 90% of intervention participants compared to 26% pre intervention. Urinalysis and testing for diabetes were not routinely conducted across either group.

### Post-birth questionnaires

Table 6 presents the scores of the Quality of Prenatal Care Questionnaire (QPCQ) [37] and the Person-Centred Prenatal Care Scale Questionnaire (PCPS) [38] and the corresponding subscales.

**Table 6:** – QPCQ and PCPS scores

	Control (*n* = 80)	Intervention (*n* = 80)
QPCQ (mean [SD])	3.61 [0.62]	3.92 [0.28]
Factor 1 – Information Sharing	3.85 [0.69]	4.21 [0.40]
Factor 2 – Anticipatory Guidance	3.01 [0.88]	3.62 [0.44]
Factor 3 – Sufficient Time	3.88 [0.84]	4.19 [0.50]
Factor 4 – Approachability	4.30 [0.62]	4.40 [0.47]
Factor 5 – Availability	2.72 [0.80]	3.00 [0.66]
Factor 6 – Support and Respect	4.02 [0.67]	4.10 [0.34]
PCPS (mean [SD])	62.93 [12.24]	68.01 [8.44]*
Communication and autonomy	54.85 [15.86]	62.11 [10.70]*
Dignity and respect	73.42 [10.08]	78.85 [8.32]*
Responsive and supportive care	60.46 [12.64]	59.74 [10.83]*

* Sample size *n* = 78

All women pre-intervention and 97.5% of the intervention group completed the questionnaires at follow-up and no questions on either scale were repeatedly omitted. However, women indicated a preference for the shorter PCPC tool [37]. Questionnaires were mostly researcher-administered (90.6%) at the woman’s home (> 90%) and completed using a tablet or phone (98.8%), with few using a paper questionnaire (1.3%). Questionnaire completion took 16–60 min. Scores for both scales were higher (favourable) in the intervention phase compared to the control, with the exception of the PCPS subscale “responsive and supportive care”. QCPQ [38] mean scores were similar between groups. The specifically designed exit questions revealed that 41 (51.3%) women believed the first antenatal visit to be too long. Seventy-seven women stated that PANDA changed the healthcare providers attitude towards them. When asked about study participation, 74 (92.6%) women in the control and 75 (93.8%) women in the intervention group rated their experience as ‘Good’ or ‘Excellent’.

### Experiences of the intervention and of research processes

Individual interviews with five health workers (intervention phase only) and 23 women (12 control phase, 11 intervention phase), and 10 non-participant observations (6 control phase, 4 intervention phase) explored acceptability of PANDA e-Health system and of research processes. An overall positive narrative of receiving and delivering ANC through PANDA emerged. Two overlapping themes were identified: *PANDA supports relational care* highlighting how PANDA helps build rapport and improve communication during consultations; and *PANDA enables systematic and personalised ANC provision* highlighting the operational advantages. Quotational evidence from women and health workers and observation extracts, are provided in Table 7., to support the main themes.

**Table 7.**
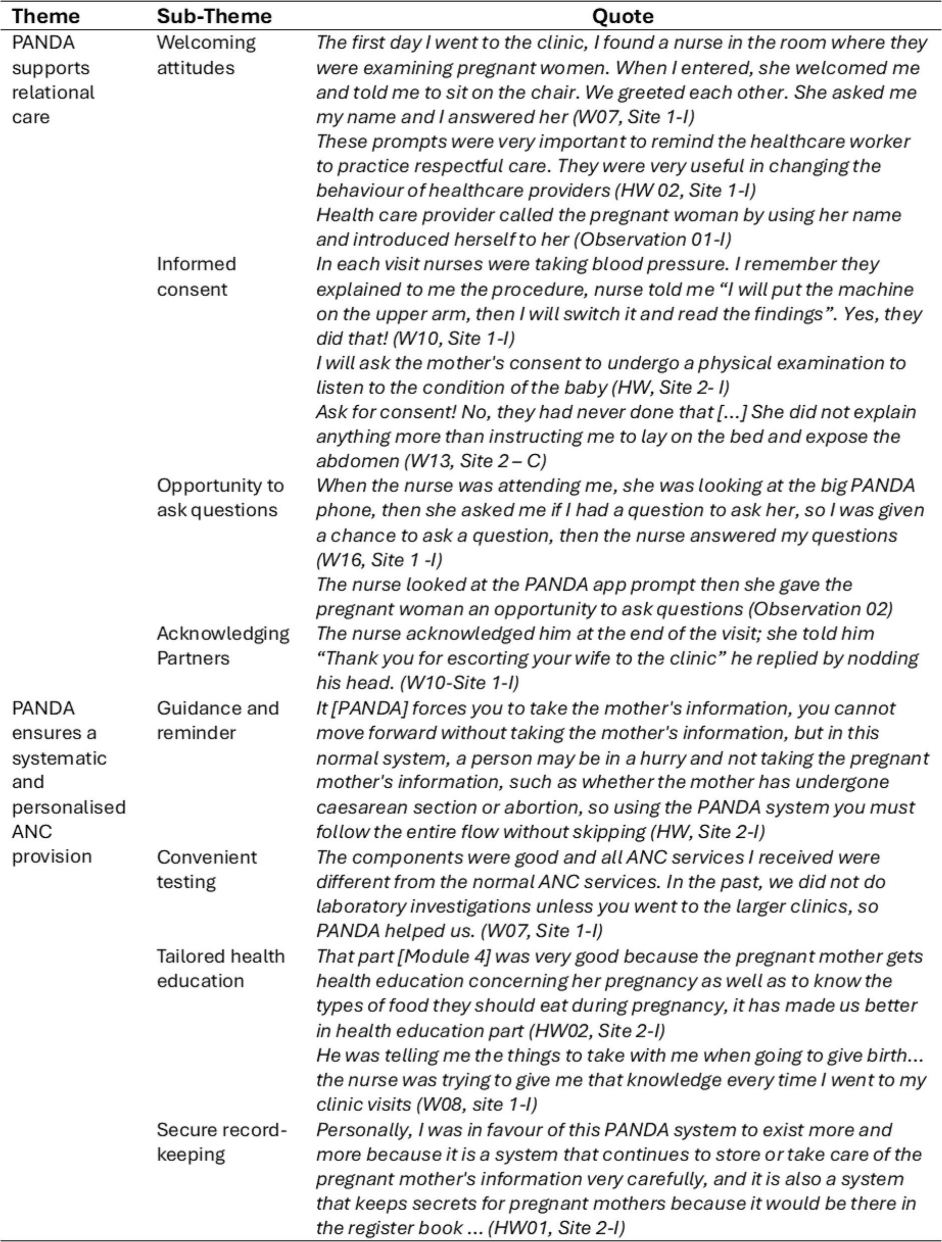
Themes

### PANDA supports relational care

#### Welcoming attitudes

All women remarked that PANDA had influenced ‘nurse’s behaviour’, including use of a respectful language, a gentle voice tone and improved body language (smile, gestures, eye contact. Being addressed by name was frequently mentioned. Some women receiving standard care, also noted positive behaviours, but this was inconsistent. Health workers also stated that their behaviour had changed because of PANDA, stated that reminders were helpful.

#### Informed consent

Prompts in PANDA were the catalyst to health workers providing women with information and seeking consent for procedures; these were welcomed by health workers. Some women compared their previous experiences, stating that the current antenatal visit was superior. Women commented on being provided with explanations about tests and examinations prior to giving informed consent. This was in stark contrast to women in the control group, who stated consent was never sought.

#### Opportunity to ask questions

At the end of each PANDA module, a prompt reminded the health worker to ask the woman if she had any concerns or questions. Being given this opportunity, several times throughout the visit, enabled verbal interactions, which built women’s confidence and trust.

#### Acknowledging partners

Women who attended ANC with their partner, after the introduction of PANDA, noticed that nurses acknowledged him and verbally appreciated that he had accompanied them. Some indicated that the nurse asked for their permission before sharing the test results and involved them during provision of health education.

### PANDA ensures a systematic and personalised ANC provision

#### Guidance and reminder

PANDA acted as guidance to provide a comprehensive service and prevented health workers from skipping or forgetting important components. For instance, collecting detailed information about previous pregnancies to identify potential risk factors and ensuring privacy and confidentiality during the visit (i.e. closing the door before starting the consultation or communicating the results to the woman). Health workers emphasised the need for training of new staff appointed during the study, and some indicated the need for more hands-on practice with PANDA.

#### Convenient testing

Point of care testing resulted in the availability of equipment, tests and medicines at the facility, and avoided women having to travel elsewhere for specific tests [confirmed through observations]. This enabled health workers to conduct a thorough assessment of women and provide medications (malaria prophylaxis, ferrous sulphate etc.) when needed. It also enabled documentation of missing tests and subsequent rationale, which was useful for stock management. Women appreciated “having everything in one place”, which negated the need to travel to other facilities, something that frequently happened during standard care provision.

#### Tailored health education

Health workers tailored the PANDA educational contents to the woman’s history and needs. Women valued receiving individualised health education, especially around nutrition and healthy practices, danger signs of pregnancy and signs of labour and how to prepare for the arrival of their child. They noticed how PANDA supported health workers in delivering these educational contents and wished for this to continue.

Some women were shown PANDA images on the tablet and stated that visualisation increased their understanding of the information and made them feel more involved in the antenatal consultation. However, for some, the positioning of their chair prevented them from seeing the images.

#### Secure record-keeping

Use of m-health was recognised as advantageous for maintaining confidentiality of women’s records and easiness of retrieving her details in subsequent appointments. This was particularly useful when a woman forgot her antenatal card.

Some challenges of PANDA were highlighted by health workers and women, primarily the duration of the first ANC visit which took between 45 and 90 min compared to the 30 to 45 min in the control. However, subsequent visits were shorter due to familiarisation with the system and less content to be covered. Sending the data to the server was sometimes an issue due to unstable internet connectivity and lack of electricity, which resulted in some frustration and the need for strategic planning. This involved using the tablet offline and transferring data when internet became available. Despite these limitations, both women and health workers wished for PANDA to continue and to be extended to other facilities.

## Discussion

We aimed to adapt PANDA to incorporate respectful care elements and assess the feasibility of conducting a large effectiveness trial to increase antenatal contacts and improve perinatal outcomes in one rural region in Tanzania. Despite the plethora of mHealth studies related to improving antenatal care [39], we are not aware of any using PANDA within a rural context in a low-income setting, such as Tanzania, that could inform a full-scale trial. We were able to demonstrate feasibility of intervention implementation and study processes, through the exceptional recruitment (> 95%) and retention (100%) rates. Furthermore, the intervention functioned well operationally and was accepted by women and health workers. We had overwhelming support to progress the research to an effectiveness study, informed by the lessons learnt.

### Strengths and limitations

This study was conducted in a rural area of Tanzania, where environmental challenges exist, related to poor infrastructure and limited transport. As 77% of stillbirths and approximately 60% of newborn deaths occur to women living in rural areas [28], it was critical that the setting reflected the need. Co-production of the PANDA system enabled the intervention to be context-specific and culturally acceptable. The feasibility study included only two dispensaries, within 20 km of the referral hospital. Inclusion of sites further away from the hospital, would have provided additional insight, but research resources limited our geographical scope. It is possible that the positive feedback from women and health workers was as a result of social desirability bias, however the high retention rates suggest PANDA was acceptable. Furthermore, interviews were conducted by researchers independent of the clinical team who delivered the intervention, and participants suggested potential improvements thus demonstrated ability to speak freely.

### Interpretation

Although earlier studies have assessed various aspects of the PANDA, and in different context [19–22], none of the studies have explored the feasibility of conducting a full-scale trial to improve clinical (perinatal) and process (antenatal visits) outcomes. This study, underpinned by the MRC/NIHR framework for complex interventions [23], provides an important exemplar of the benefits of adequate developmental work, prior to embarking on resource-intensive effectiveness trials. Furthermore, it describes the intervention development process, something which is often underreported [40].

Critically, we gained understanding of the context in which the intervention would be implemented and involved key stakeholders and community members in the adaptation of PANDA, to optimise its acceptance and useability. Although generally considered acceptable, the development work enabled us to improve the intervention in several ways, including cultural adaptation of the content, use of local language, and inclusion of mental health elements. Going forward, the need for additional hands-on training and addressing the challenge of the duration of the first visit would also be important. For the latter, preparing those using PANDA, and incorporating this time into clinic schedules would be essential.

Having the ability to conduct point of care testing was critical to adequate antenatal assessments. Therefore, we ensured that materials were available at sites to carry out any investigations. When developing a protocol for a full-scale evaluation, one would have to consider whether point of care testing materials should be provided, and how they would be sustained. Furthermore, one may need to consider whether any differences are in fact due to point of care testing, the PANDA application or a combination of both.

One of the most important adaptations was incorporating respectful care into the PANDA application to initiate health worker behaviour changes. Having identified aspects of disrespectful antenatal care in the preparatory phase, it was critical that this was addressed as part of the intervention. We know, from a number of studies, that disrespectful care can prevent women from accessing antenatal services [10] and/or deter them from returning [41]. Poor attendance rates can lead to morbidity and mortalities [42], and lack of birth preparedness [7]. Whilst other studies have used mHealth to target behaviour modifications [22], integrating the behaviour prompts alongside other screening items within the application provided a seamless, and real-time change. Health workers welcomed the reminders to provide respectful care and were observed doing so. The prior training and mentorship, provision of digital tablets, and motivation to improve care, are likely contributors to the behaviour change, with the potential for habit formation.

We learnt several lessons related to the research process. In terms of recruitment, the partners agreement was necessary prior to consent, information had to be supplied in local language, and visualisation of the intervention supported intervention acceptance. Follow-up was resource-intensive, requiring adequate manpower and community engagement, suggesting that a larger study would require a different approach, particularly as almost 40% of participants birthed at home. Obtaining alternative contact details and using remote follow-up are potential solutions. Identifying participants’ preference for the shorter questionnaire and for it to be researcher administered were also important.

## Conclusion

Acceptability of PANDA use and feasibility of study processes were demonstrated. Participants’ and stakeholders’ feedback have informed refinement of the PANDA package and its implementation strategy ahead of commencing a cluster trial to assess effectiveness.

## Data Availability

The datasets analysed during the current study are available from the corresponding author on reasonable request.
